# Mesothelin as a novel biomarker and immunotherapeutic target in human glioblastoma

**DOI:** 10.18632/oncotarget.20303

**Published:** 2017-08-16

**Authors:** Zhenjiang Liu, Martin Rao, Thomas Poiret, Silvia Nava, Qingda Meng, Anna von Landenberg, Jiri Bartek, Shanshan Xie, Georges Sinclair, Inti Peredo, Ernest Dodoo, Markus Maeurer

**Affiliations:** ^1^ Department of Laboratory Medicine, Division of Therapeutic Immunology, Karolinska Institutet, Stockholm, Sweden; ^2^ Centre for Allogeneic Stem Cell Transplantation, Karolinska University Hospital Huddinge, Stockholm, Sweden; ^3^ Department of Neurosurgery, Karolinska University Hospital, Stockholm, Sweden; ^4^ Department of Clinical Neuroscience and Department of Medicine, Karolinska Institutet, Stockholm, Sweden; ^5^ Department of Neurosurgery, Copenhagen University Hospital Rigshospitalet, Copenhagen, Denmark; ^6^ Department of Microbiology, Tumor and Cell Biology, Karolinska Institutet, Stockholm, Sweden

**Keywords:** glioblastoma, mesothelin, T-cell response, interferon gamma, immunotherapy, Immunology and Microbiology Section, Immune response, Immunity

## Abstract

Glioblastoma multiforme (GBM) presents the most malignant form of glioma, with a 5-year survival rate below 3% despite standard therapy. Novel immune-based therapies in improving treatment outcomes in GBM are therefore warranted. Several molecularly defined targets have been identified mediating anti-GBM cellular immune responses. Mesothelin is a tumor-associated antigen (TAA) which is expressed in several solid tumors with different histology. Here, we report the immunological significance of mesothelin in human malignant glioma. Expression of mature, surface-bound mesothelin protein was found to bein human GBM defined by immunofluorescence microscopy, and on freshly isolated, single cell suspension of GBM tumor cells and GBM tumor cell lines, determined by based on flow cytometric analysis. Peripheral blood (PB) from patients with GBM, stimulated with mesothelin peptides and IL-2, IL-15 and IL-21, exhibited increased antigen-specific IFN-γ and TNF-α production. Anti-mesothelin directed T-cell responses could also be detected in tumor - infiltrating lymphocytes (TIL) isolated from GBM speciments. Furthermore, T cells cultured in the presence of IL-2, IL-15 and IL-21 displayed enhanced mesothelin-specific CD4+ and CD8+ subset proliferation, based on ELISA and flow cytometric readouts. Mesothelin-specific IgG antibodies as well as (shed) mature mesothelin protein were detected in plasma samples from patients with GBM by indirect ELISA. Finally yet importantly, we identified distinct immune recognition hotspots within the mature mesothelin component, defined by peptide-specific IFN-γ responses from peripheral T-cells from patients with GBM. Mesothelin may therefore qualify as a viable target for immunotherapeutic approaches for patients with GBM.

## INTRODUCTION

Glioma represents the most severe form of a CNS malignancy arising from a mutation-driven pathological transformation of glia cells in the brain [1]. Gliomas manifest in several histological forms, depending on stage of disease: A: diffuse or anaplastic astrocytoma (IDH-wildtype/-mutant/not otherwise specified (NOS)); OD: oligodendroglioma or anaplastic oligodendroglioma (IDH-mutant and 1p/19q-codeleted/NOS); OA: oligoastrocytoma or anaplastic oligoastrocytoma (NOS); GBM: glioblastoma multiforme (IDH-wildtype/-mutant/NOS) [2, 3]. The most malignant of these is GBM, representing 55% of all glioma diagnoses in humans. Up to now, the survival rate for individuals diagnosed with GBM is very poor - only 3% of patients exhibit a 5-year survival rate [4]. Low-grade astrocytomas can eventually progress to GBM, at which stage treatment options become limited. While mutant forms of the oncoprotein p53 as well as isocitrate dehydrogenase (IDH) are found in many low-grade gliomas [1, 2], mutations in epidermal growth factor receptor (EGFR) is present in 40% of GBM cases, the most common being the EGFRvIII mutation, which seen in approximately 25% of GBM [5].

The standard treatment for GBM comprises radiotherapy in conjunction with adjuvant temozolomide, an oral alkylating agent which has proven anti-tumor effects [2]. Most patients undergo surgical resection of the tumor, although the prognosis is still rather dark, i.e. 17 months survival post-surgery, most patients experience tumor recurrence [6]. Salvage therapy with Gamma Knife Surgery (GKS) for selective removal of the tumor (as opposed to whole-brain radiotherapy) has been shown to modestly improve the median survival of patients with recurrent GBM, particularly when administered adjunctively to standard chemotherapy and immunotherapy (anti-vascular endothelial growth factor (VEGF) and a monoclonal antibody, bevacizumab (Avastin^®^) [6]. Nevertheless, a smaller reduced tumor volume, the rate of diffusion and response to adjuvant therapy may increase GKS efficacy and subsequent treatment outcome for patients. Cell-based therapies, targeting cancer - associated antigens, are being studied for reduced toxicity, improved therapeutic performance and extended life span of patients with malignant gliomas [7].

Mesothelin is a 40 kDa tumor differentiation antigen present on normal mesothelial cells, but overexpressed in mesothelioma, meningioma, ovarian cancer, lung cancer and pancreatic adenocarcinomas [8–11]. The unprocessed mesothelin precursor comprises two components, namely the 31 kDa megakaryocyte-promoting factor (MPF) and the membrane-anchored, 40 kDa mesothelin-glycoinositolphoslipid (GPI) component [8]. MPF is cleaved by furin and shed into systemic circulation, and has been evaluated as a more accurate biomarker, as compared to the mesothelin precursor for the immunodiagnosis of mesothelioma [12]. Cell membrane-bound (or mature) mesothelin selectively binds to mucin 16 (MUC16), which is expressed in the peritoneum, pleural cavities, mucosal surfaces and the brain [13]. The expression of mesothelin in meningioma lead to the question whether the antigen is also expressed in malignant brain tumours, particularly GBM. In addition, a 2014 study showed that CNS metastasis is highly likely in patients with triple negative breast carcinoma who are mesothelin positive at diagnosis (63%) [14]. Mesothelin overexpression in approximately 50% of advanced gastroesophageal cancers was recently reported, where expression levels were shown to increase with tumour staging [15]. However, no formal link between mesothelin and primary malignant gliomas in humans has been establishedat this point.

Using a combination of histological and immunological approaches, we present here for the first time the correlation between mesothelin expression in GBM and clinical outcome. We also describe mesothelin as a hitherto unknown biomarker for immunodiagnosis as well as a viable target for the immunotherapy of GBM.

## RESULTS

### Mature mesothelin protein is expressed in GBM tissue and tumor cells

**Figure 1:**
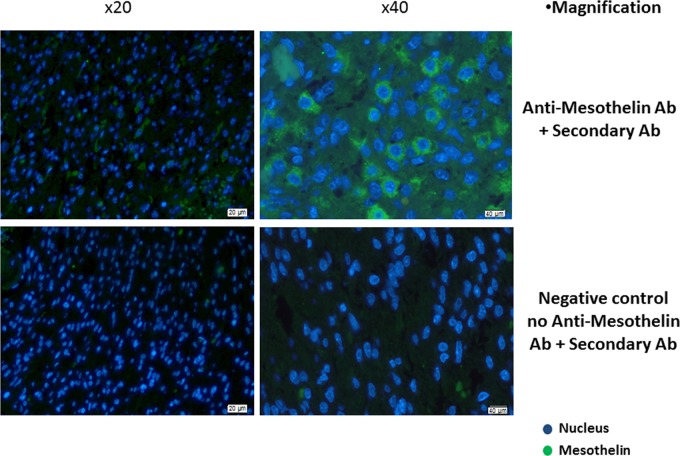
Immunohistological confirmation of mesothelin overexpression in GBM tissue Paraffin-embedded tissue sections were stained with rat anti-human mesothelin primary antibody. Detection was performed using a goat anti-rat IgG secondary antibody labelled with Alexa Fluor 488-conjugated polyclonal goat anti-rat secondary antibody. The primary antibody was omitted in the negative control while the pancreatic cancer tumour cell line PaTu was used as positive control (Supplementary Figure 1). Shown are representative photographs for GBM tissue from one patient.

We confirmed the expression of mesothelin in GBM tissue from 4 out of 11 patients (36.4%) by immunohistological staining of mesothelin protein in paraffin-embedded tissue sections visualized by fluorescence microscopy (representative image of mesothelin immunostaining in GBM tissue from one patient is shown in Figure 1). As a negative control, we also immunostained tissue sections from the same tissue block exclusively with the secondary antibody, and observed that the binding of the anti-mesothelin primary antibody was target-specific (Figure 1 & Supplementary Figure 1). We also performed a flow cytometric assay to confirm surface expression of mesothelin on GBM tumor cells (freshly isolated viable tumor cellscultured *in vitro*) from five patients, in addition to known GBM tumor cell lines as controls i.e. SNB19 and DBTRG05. The K562 chronic myelogenous leukaemia cell line was used as a negative control [16], while the PaTu pancreatic cancer cell line served as positive control for mesothelin expression. Three out of five cell lines from patients with GBM exhibited positive specific staining for mesothelin,; co-staining with a glia cell -specific marker exhibited co-staining of anti-mesothelin - reactive cells thus enabling us to conclude that mature mesothelin is a viable biomarker for human GBM tumors (Supplementary Figure 2).

### WBA IFN-γ responses of patients with brain tumour to the mesothelin precursor, MPF and mesothelin proteins

**Figure 2:**
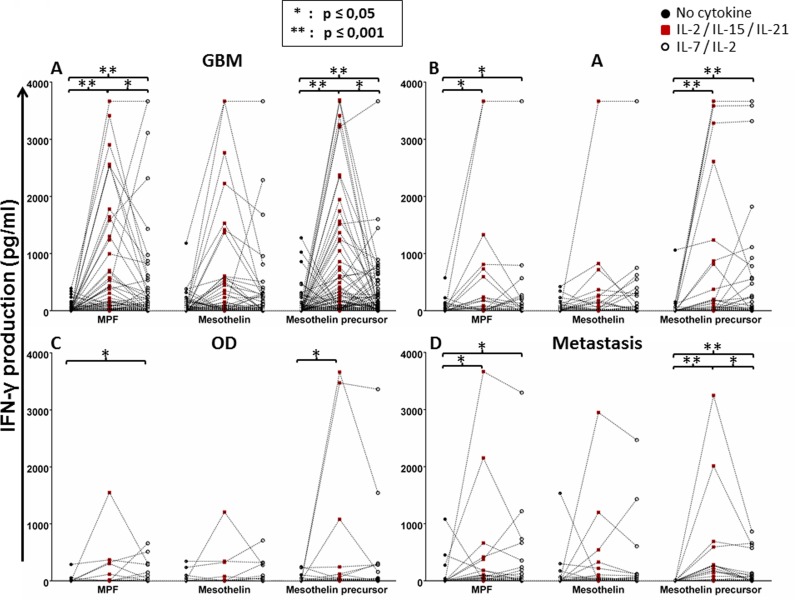
Whole-blood IFN-γ responses of glioma patients to the mesothelin precursor protein, MPF and the mature mesothelin component with or without cytokine conditioning Whole-blood obtained from patients with glioma (GBM, *A*; astrocytoma, *B*; OD, *C*; metastasis, *D*) were cultured with mesothelin or its derivatives in the absence of cytokine conditioning, with IL-2/IL-7 or IL-2/IL-15/IL-21 conditioning over seven days. Supernatants were then harvested for IFN-γ detection by ELISA. Shown are dot plots representing responses of individual patients. Mann Whitney test of medians was performed to gauge statistical significance. *p < 0.05; **p < 0.001.

We found that conditioning of whole blood from patient with GBM with IL-2, IL-15 and IL-21 significantly improved the IFN-γ response to the mesothelin precursor peptide pool, as well as the MPF subcomponent (Figure 2A). The addition of IL-2 and IL-7 to the antigen-PBMC cultures resulted in increased IFN-γ responses to all three mesothelin-associated targets i.e. peptide pools spanning the precursor mesothelin molecule, MPF and mature mesothelin, respectively although to a lesser degree as compared to co-culture with IL-2, IL-15 and IL-21. The finding was similar for patients diagnosed with A, OD or patients with metastatic brain tumosr (M) with regard to IFN-γ responses to the mesothelin precursor and MPF components, respectively comparing IL-2 / IL- 7 with with IL-2, IL-15 and IL-21 conditioning (Figures 2B-2D). Although not statistically significant, the IFN-γ response to the mesothelin peptide mix was improved by adding IL-2, IL-15 and IL-21 as compared to exposure to the respective target antigens, or by adding IL-2 and IL-7. When the patient groups (different histology and grade of glioma) were compared to each other, the adding of cytokines was associated with an increased production of IFN-γ to the mesothelin precursor, MPF or mesothelin components respectively. However, we were only able to identify statistical significance for mature mesothelin-specific T-cells responses in the absence of additional cytokine ‘conditioning’ of the T-cell responses (Supplementary Figure 3A). As a control, we also tested the recognition of precursor mesothelin, MPF and mature mesothelin in in PBMCS from peripheral blood of healthy donors, and found antigen-specific IFN-γ only in 1/3 donors, yet exclusively if PBMCs were expanded with with IL-2, IL-15 and IL-21 conditioning but not with IL-2 and IL-7 treatment, or in the absence of added cytokines conditioning (Supplementary Figure 3B). Thus, we concluded that robust IFN-γ responses directed against the mesothelin precursor and its derivatives can be amplified by adding IL-2, IL-15 and IL-2 to T-cell-target cell cultures.

### Tumour grade-associated WBA IFN-γ responses of patients with brain cancer to mesothelin precursor and its derived antigenic components

**Figure 3:**
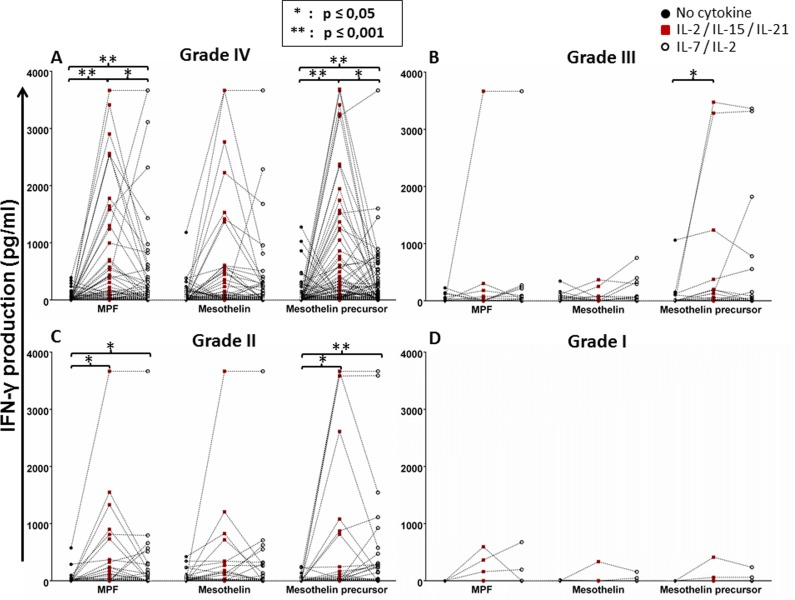
Whole-blood IFN-γ responses to the mesothelin precursor protein and its derivatives with or without cytokine conditioning based on glioma grading The data presented in Figure 1 was reanalysed based on WHO tumour grading, grade I being the least malignant and grade IV being the most aggressive (see ‘Results’). Shown are dot plots representing responses of individual patients. Mann Whitney test of medians was performed to gauge statistical significance. *p < 0.05; **p < 0.001.

Next, we reanalysed the IFN-γ response data in relation to the WHO brain tumour grading system, as the severity of disease directly influences the ‘immunological fitness’ of the patient [1, 3]. According to this system, there are four grades of brain tumors in humans: grade I tumors are benign and linked with long-term survival (i.e. pilocytic astrocytoma) which were employed as internal control; grade II tumors are slow-growing with metastatic potential (i.e. diffused astrocytoma); grade III tumours are malignant and have a chance to recur at the same grade or progress to grade IV (i.e. anaplastic astrocytoma); grade IV tumours are the most malignant and can metastasise rapidly (exclusively GBM). In the reanalysis, we found that patients with grade IV brain tumour (GBM), constituting the majority of group of the study cohort, exhibited a significantly increased IFN-γ response to MPF and mesothelin precursor peptide pool if the PBMCS were conditioned with IL-2/IL-15/IL-21 (Figure 3A). A somewhat similar pattern was also true for patients with grade II brain tumors (Figure 3C). Since only very few patients had a diagnosis of grade I or III brain tumours, a strong effect of cytokine conditioning on IFN-γ response to the mesothelin antigens could not be seen (Figure 3B, 3D). Nevertheless, patients with grade III brain tumors may still have a benefit, due to a significant improvement in their IFN-γ response to the mesothelin precursor molecule (Figure 3B). When patient groups, based on the different grades of CNS tumors were directly compared to each other, there appeared to be an increased production of IFN-γ to the mesothelin precursor, MPF or mesothelin components with cytokine conditioning, respectively albeit without reaching statistical significance (Supplementary Figure 4). We therefore concluded that the mesothelin-specific response of patients with grade IV brain tumors has potential to be enhanced with IL-2/IL-15/IL-21 conditioning.

### Flow-cytometric assay of specific cell-mediated immune response in activated whole blood (FASCIA) analysis of T-cell proliferation

**Figure 4:**
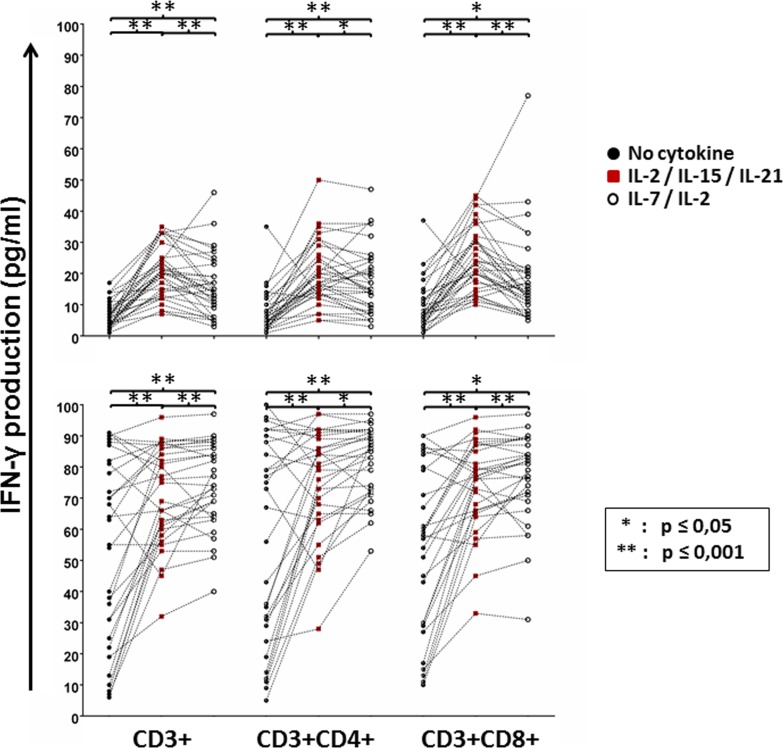
GBM peripheral blood T-cell proliferation in response to mesothelin stimulation with or without cytokine conditioning Immune cells remaining in the culture plate at the end of the whole blood assay were purified, processed and stained with CD3, CD4 and CD8 antibodies for flow cytometric analysis Shown are dot plots representing responses of individual patients. Mann Whitney test of medians was performed to gauge statistical significance. *p < 0.05; **p < 0.001.

In order to investigate whether the ability of the patients’ cells to produce IFN-γ in response to antigen stimulation corresponded with enhanced proliferation of T-cells in the WBA, we measured the numbers of CD3+, CD3+CD4+ and CD3+CD8+ cells using flow cytometry. As expected, conditioning with IL-2/IL-15/IL-21 significantly increased the numbers of total (CD3+), CD4+ and CD8+ T cells in response to the mesothelin precursor (Figure 4A). However, compared to IL-2/IL-15/IL-21 conditioning, the addition of IL-2/IL-7 to T-cell cultures appeared to have a more pronounced effect on T-cell proliferation in response to PHA stimulation, although both cytokine cocktails markedly improved cell growth (Figure 4B). Thus, conditioning with IL-2/IL-15/IL-21 amplifies the antigen-specific cellular immune response to the mesothelin precursor by inducing T-cell proliferation.

### Mesothelin peptide-specific WBA IFN-γ responses

**Figure 5:**
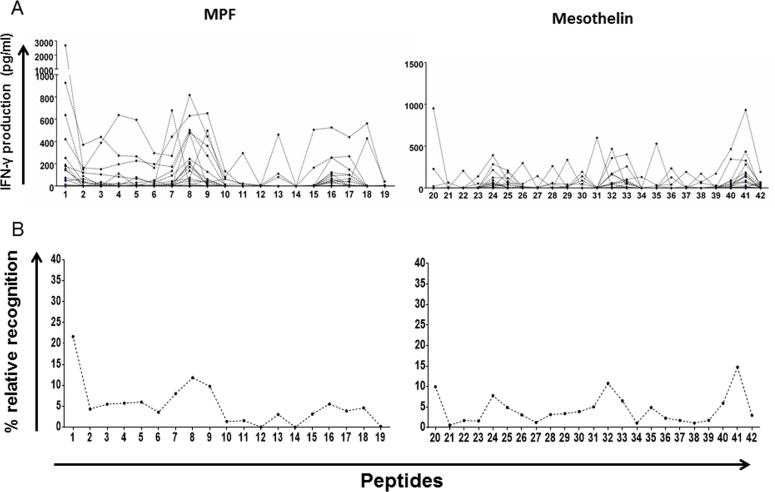
Whole-blood IFN-γ responses to peptides spanning the MPF and mature mesothelin components 42 non-overlapping 15-mer peptides spanning the entire mesothelin molecules were exposed to peripheral blood of GBM patients over a seven-day period. Supernatants were then harvested for IFN-γ detection by ELISA. Absolute IFN-γ concentrations (pg/ml) produced by each patient for a single peptide **A.** as well as the average value per peptide (total IFN-γ/no. of patients) normalised to the sum of IFN-γ production for the entire peptide pool in percentage **B.** are shown. Immune hotspots within the mesothelin component, defined by peptide-specific IFN-γ production, identify several peptides which may represent viable targets to expand T-cells in host-directed therapies.

After we had confirmed that patients with brain cancer can mount a measurable cellular immune responses to the mesothelin precursor, we wanted to map which epitopes within the mesothelin protein would evoke the strongest IFN-γ response by T cells. We used a pool of 42 chemically synthesized peptides spanning the entire mesothelin precursor molecule for the T-cell recognition mapping. The first 19 peptides comprise the MPF component, while the following 23 peptides constitute the (mature) mesothelin domain. We plotted the absolute values for IFN-γ production per patient, as well as the percentage of normalised average response ((IFN-γ response to a single peptide/IFN-γ response to entire mesothelin peptide pool)/100)) - which provides an indication of the magnitude of the T-cell responses directed against a single peptide (potentially epitope) across the entire protein (relative recognition). Immune response ‘hotspots’ were detected within MPF as well as the mature mesothelin peptides (Figure 5A), although a a high number of epitopes, which induced stronger IFN-γ production among the patients, were found in the mature mesothelin domain, with PBMCs from two patients recogniszing and strongly responding exclusively to only a single mesothelin epitope (Supplementary Figure 5), we also We also found, after calculating the relative recognition based on the normalised average response (%), that several clear immune recognition hotspots for T-cell recognition existed in the mature mesothelin component that may drive the mesothelin-directed cellular immune response (Figure 5B). Thus, the mapping of the mesothelin domain callowed to describe peptides that represent potentially strong T-cell targets for T-cells from patients with CNS tumors.

### Mesothelin-specific IgG in plasma from patients with GBM

**Figure 6:**
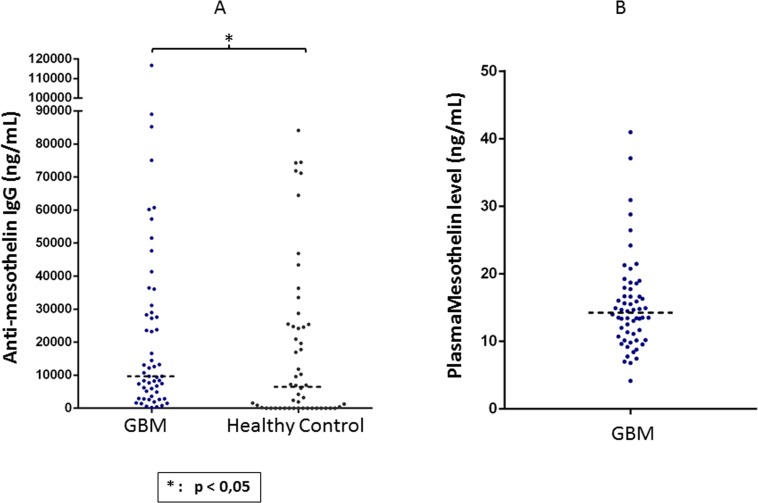
Detection of mesothelin-specific circulating IgG as well as shed mesothelin protein in plasma of GBM patients An indirect ELISA method was used for quantifying mesothelin-specific IgG titres in plasma, while a commercially available ELISA kit was used for measuring mesothelin protein. IgG titres and mesothelin levels are expressed as ng/ml of plasma. Mann Whitney test of medians was performed to gauge statistical significance. *p < 0.05; **p< 0.001.

Since humoral immune responses are clinically significant in cancer, we quantified theamount of mesothelin-specific antibodies in serum obtained from patients with GBM as well in serum from healthy individuals. We found that patients with GBM had significantly higher titers of mesothelin-specific circulating IgG as compared to healthy individuals (Figure 6A). We also measured the amount of soluble mesothelin precursor in plasma of patients with GBM, where the median concentration was approximately 15 ng/ml (Figure 6B). Thus, Hassan and colleagues previously showed that serum mesothelin is detectable in patients with mesothelioma or in serum, from patients with ovarian cancer at a significantly higher median concentration as compared to serum from healthy donors [17].

### Mesothelin-specific response of GBM TIL

**Figure 7:**
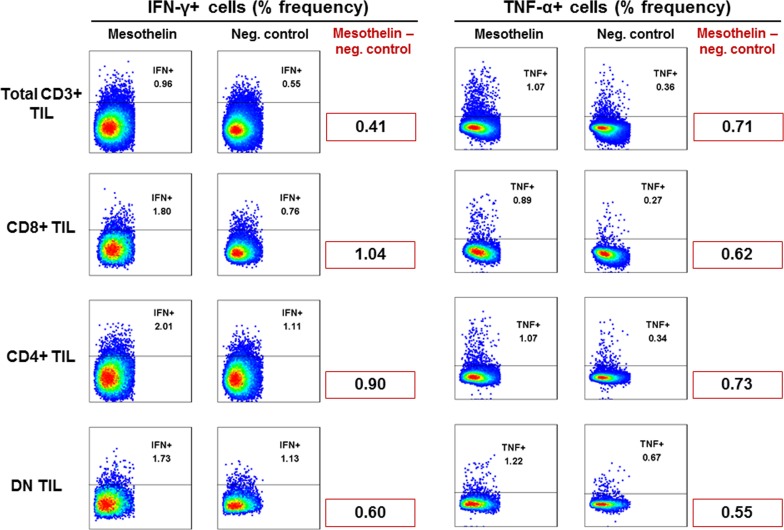
Recognition of mesothelin peptides by GBM TIL TIL isolated from GBM tumor tissue were cultured *in vitro* with IL-2/IL-15/IL-21 and exposed to mesothelin peptides over a 6-hour period. Cells were then stained with CD3, CD4, CD8, IFN-γ and TNF-α to visualise intracellular cytokine production by mesothelin-specific T-cells. Shown are frequencies (%) of responding cells based on CD3+ TIL of one representative patient. Cell frequency of more than 0.2% is a considered a legitimate response.

TIL from patients with GBM were tested for reactivity to mesothelin measured by intracellular cytokine production of IFNγ and/or TNFα. CD4+ as well as CD8+ TIL produced a measurable cytokine response, particularly CD8+ TIL produced IFNγ, while CD4+ TIL appeared to be the source for TNF α production (Figure 7). Double - negative T-cells (i.e. CD3+, CD4-, CD8-) reacted as well with TNFα and IFNγ production. We therefore concluded that tumor-infiltrating lymphocytes harvested from patients with GBM contain T-cells that are able to react to mesothelin.

## DISCUSSION

Research in cancer biomarkers and druggable targets in patients with GBM is clinically needed due to the dire prognostic factors for patients with CNS tumors. Pertinent to glioma, the type III epidermal growth factor receptor (EGFRvIII) protein has been a major target for immunotherapies, especially for the development of specific chimeric antigen receptor (CAR) T cells [18]. Nevertheless, newer therapeutic targets for fatal malignancies such as GBM are necessary in order to expand the current treatment options. In this study, we describe for the first time the tissue expression and immunological recognition pattern of mesothelin in patients with gliomas. To the best of our knowledge, mesothelin has not been studied in the context of WHO grade IV gliomas (GBM) in humans. The closest link between mesothelin and neuropathology in humans has been the observation that mesothelin is expressed in meningeal arachnoidal cells that may drive malignant transformation in meningioma [19]. Using immunofluorescence microscopy, we could visualise that the mesothelin protein is overexpressed in GBM tissue samples. Furthermore, our immunological data suggests that immune cells from patients with malignant primary glioma (i.e. GBM) can strongly recognize and respond to cell surface-bound, mature mesothelin (GPI-anchored component) *via* cytokine production (IFN-γ, TNF-α), as well as antibody (IgG) production T cells from patients with GBM are able to expand dramatically in the presence of conditioning medium containing IL-2/IL-15/IL-21 as well as the mesothelin peptide pool - for antigen-specific cell activation. This also applies to TILs from patients with GBM or pancreatic cancer [20]. We also We were not able to detect a ‘baseline’ immune reactivity directed to mesothelin peptides in T-cells obtained from peripheral blood from healthy donors, in line observed that with peripheral blood from healthy donors, there was no baseline induction of IFN-γ production in response to mesothelin peptides, in line with previous findings [21]. However, with IL-2/IL-15IL-21 conditioning, there appeared to be a dramatic increase, in mesothelin reactivity suggesting that there are indicating that there are circulating mesothelin-specific T cells in peripheral blood of individuals who are cancer-/tumor-free, although these anti-mesothelin - reactive T-cells are below detection levels. This is clinically relevant from an immunotherapy viewpoint, since Stronen and colleagues recently showed that HLA-matched donor-derived TCRs, that recognize private mutated cancer epitopes (neoepitopes) from patients with cancer, can be transduced into patient-derived T cells for adoptive therapy [22].: anti-mesothelin - reactive T-cells could potentially expanded from the precursor T-cell pool from healthy donors. As we have previously shown, the IL-2/IL-15/IL-21 cocktail is able to preferentially trigger expansion of antigen-specific, highly activated CD8+ TIL, that are not immunologically exhausted and are capable of killing autologous GBM cells *in vitro*. Furthermore, detection of mesothelin-specific IgG antibodies in plasma, as well as the antigen mesothelin itself affirms that activation of cellular and humoral immune responses directed against mesothelin is a previously unknown characteristic of patients with GBM.

We also observed mesothelin peptide-specific T-cell IFN-γ responses in peripheral blood from patients with GBM, and identified several immune recognition ‘hotspots’ within the mesothelin precursor molecule - particularly in the mature mesothelin component. Importantly, the mesothelin reactivity of peripheral blood lymphocytes is a strong indication of the presence of distinct populations of circulating epitope-specific T cells that express adequately responsiveness TCRs. Equally important is that the mesothelin-specific T-cell responses in peripheral blood can be amplified with a combination treatment of IL-2, IL-15 and IL-21. These observations highlight the potential T-cell epitopes of mesothelin that could be used to develop targeted cell-based therapies to achieve clinically relevant immune responses. Expansion of mesothelin - directed T-cells may require further validation, since the restricting HLA elements of the CD8 and/or CD4 subsets recognizng mesothelin peptides need to be ascertained for individual patients in order to select patients who may benefit most from a - mesothelin - (MHC class I- or class II - restricted) immunotherapeutic approach.individuals the most robust, as earlier studies showed that Immune monitoring studies for clinical trials of a GM-CSF-overproducing allogeneic pancreatic cancer cell line-based vaccine showed that patients with pancreatic adenocarcinoma, who were treated with an GM-CSF producing allogeneic cancer cell line, exhibited a consistent induction of HLA-A1- and A2-restricted CD8+ T-cell responses to mesothelin epitopes marked by IFN-γ production and/or cytotoxic activity [23–25].

In addition, whether mutated mesothelin, like EGFRvIII, is also involved in GBM disease progression and recognition by GBM TIL as well as peripheral blood lymphocytes remains to be determined since there have been no previous studies concerning mutant mesothelin protein and pathology and/or disease progression in human cancers. The association between anti-programmed cell death 1 (PD-1) therapy and activation of CD8 T cells specific for mutated antigens in melanoma [26, 27] may warrantstudies with mutated mesothelin-specific responses in patients with GBM undergoing immune checkpoint blockade therapy [28].

The overexpression of mesothelin is associated with particularly poor prognosis for patients with lung adenocarcinoma harbouring mutations in the v-Ki-ras2 Kirsten rat sarcoma viral oncogene homolog (KRAS) gene [29, 30]. Furthermore, the oncogenic version of KRAS protein, KRAS G12X, which is associated with aggressive disease in human cancer including malignant glioma [30–34], was shown to promote initiation and rapid tumour progression in a zebrafish model of human malignant glioma [35]. Whether the overexpression of mesothelin in human GBM also influences aberrant expression of other oncogenic genes and/or proteins is currently being examined in our laboratory. These results are expected to shape future clinical studies to test targeting multiple cancer epitopes simultaneously. in order to avoid potential antigen-loss variants in the course of immunological therapies.

Several mesothelin-based biological therapies i.e. chimeric antigen receptor (CAR T cells), monoclonal antibodies, antibody-drug conjugates are currently under clinical assessment in patients with advanced solid tumours, such as mesothelioma, lung, pancreatic and ovarian cancer [36, 37]. In fact, a recently published clinical case report showed that mesothelin-directed CAR T cells were able to mediate tumor regression in two patients; the first with malignant pleural mesothelioma and the second patients with pancreatic ductal adenocarcinoma [38]. Based on our findings and evidence in literature, we are inclined to speculate while mesothelin overexpression may promote GBM pathology, that cellular and humoral immune responses directed against mesothelin this molecule may also trigger potent anti-tumor immune responses, presenting a novel antigenic target for immunodiagnosis and immunotherapy - with respect to antibody- and cell-based approaches for patients with GBM.

To conclude, this is the first study to describe mesothelin as an immunologically relevant target in human GBM. Mesothelin is able to induce potent cellular as well as humoral immune responses in patients with GBM, in addition to being present at measurable amounts in systemic circulation. We also show that preconditioning peripheral blood lymphocytes with a combination of the gamma chain cytokines IL-2, IL-15 and IL-21 will amplify the anti-mesothelin cellular immune response. Clinical phase I trials are now warranted in order to develop cellular therapies for human CNS cancers using mesothelin as a target.

## MATERIALS AND METHODS

### Patient characteristics/cohort description

The regional ethical review board (Regionala etikprövningsnämnden) at Karolinska Institutet, Stockholm, Sweden, approved this study (diary number: 2013/576-31). Patients with brain cancer (*n* = 286) registered at the Karolinska University Hospital, Stockholm were recruited following written informed consent. The patients were diagnosed with the following forms of brain cancer: A: diffuse or anaplastic astrocytoma (IDH-wildtype/-mutant/not otherwise specified (NOS)); OD: oligodendroglioma or anaplastic oligodendroglioma (IDH-mutant and 1p/19q-codeleted/NOS); OA: oligoastrocytoma or anaplastic oligoastrocytoma (NOS); GBM: glioblastoma multiforme (IDH-wildtype/-mutant/NOS); Met: metastatic brain tumor. All participating patients were chemotherapy-naïve and had not undergone surgery. The tumours were graded according to the WHO grading system for tumours of the central nervous system [2]. The clinical characteristics of the study cohort is summarised in Table 1. Peripheral blood samples were collected in heparin-containing tubes by venepuncture and processed in the laboratory within 24 hours. GBM tumour tissue samples were placed in Cellgro medium (Cell Genix, Freiburg, Germany) containing 10% human AB serum (Innovative Research, Michigan, USA) and processed within 3 hours.

**Table 1:** Clinical characteristics of the patient cohort

Patient characteristics	Malignant Glioma						Metastasis
	Histology			Grade			
	GBM	A	OD	IV	III	II	
**Sample Size(N)**	169	45	27	169	20	52	45
**Age Median(Years)**	62	34	40	62	48	36	60
**Age Range(Years)**	16-80	20-75	22-62	16-80	20-72	22-76	30-84
**Sex(Male/Female)**	111/58	33/12	14/13	111/58	13/7	32/20	21/24

GBM: Glioblastoma Multiform, A: Astrocytoma, OD: Oligodendroglioma.

### Mesothelin immunofluorescence microscopy

Formalin-fixed and paraffin embedded brain tissue sections were deparaffinised by washing two times, 10 minutes each, in xylene and progressively rehydrated from 100% to 50% ethanol, rinsed in water and placed in PBS for 10 minutes. All washing steps between incubations were performed in PBS. Before blocking and incubation with the primary antibody, tissue sections were subjected to heat-induced epitope retrieval using Antigen Retrieval Reagent-Basic (R&D systems, Minnesota, USA). Sections were washed in PBS and blocked with 1% normal goat serum in PBS for 30 minutes. Incubation with rat monoclonal anti-human mesothelin antibody, 10μg/ml (R&D systems, Minnesota, USA) was performed overnight at 40°C. As a negative control, the primary antibody was omitted and as positive control, pancreatic cancer tumour cell line (PaTu) was used. After washing, tissue sections were incubated for 30 minutes with Alexa Fluor 488-conjugated polyclonal goat anti-rat secondary antibody (Thermo Fisher Scientific, Massachusetts, USA) at 5μg/ml. Sections were DAPI stained after washing, and mounted in mounting medium containing anti-fade. The stained tissue sections were examined and photographed with a camera-equipped microscope (Olympus BX51, Tokyo, Japan).

### Flow cytometric analysis of mesothelin expression on tumour cells surface

#### Glioma

Ttumor tissue samples were obtained from patients with glioma is harvested byundergoing brain brain surgery inat the Department of Neurosurgery, Karolinska University hHospital, Solna, Stockholm, and then transferred toin in Cellgro GMP Serum-free Dendritic Cell Medium (CellGernix, Freiburg, Germany) plus withand 5% pooled human AB serum (Inonative Research, Michigan, USA). Then GBM tuThe tissue was cut into small fragments ofmor tissue was dissected into fragments (approximately 1-2mm^3^ in size) byusing a sterile scalpel or processed into a tissue homogenate byusing a BD Medimachine (Beocton, Dickinson, CA. USA), followed by a 2X. Wwash the tissue fragments or homogenate 2 times wiwith PBS. The processed tissue material was then culture in T75 flasks incontaining RPMI 1640 L-glutamine (2mM) medium with antibiotics (penicillin,100IU/mL and streptomycin, 10mg/mL) (Life Technologies, Carlsbad, USA) plusand 20% Foetal Bovine Serum, FBS (gGlibco, MassachusettesMassachusetts, USA). The tumor cells would settingled down and down and attached toto the bottom surface of the flask grow within 3-4 days;. Change medium change and split tumor cellsculture splitting in to different flask when necwas implemented as necessary. The following Hhuman tumor cell lines were purchased from bought from ATCC (the American tType cCulture cCollection (ATCC): include Hela (cervical cancer), DBTRG05 (GBM), SNB19 (GBM), Pa-Tu (pancreatic adenocarcinoma) and K562 (chronic myelogenous leukaemia), and were cultured in T75 flask in RPMI 1640 L-glutamine (2mM) with antibiotics (penicillin,100IU/mL and streptomycin, 10mg/mL) (Life Technologies, Carlsbad, USA) plus 20% Fetal Bovine Serum (glicocultured under identical conditions to the primary glioma tumor cells., Massachusettes, USA). The cultured tumor cellsTumor cells from glioma t (primary glioma asas well asumor tissue or form tumor cell lines) were collectedharvested and washed once with PBS once, thenand stained stained with the following fluorochrome-conjugated monoclonal antibodies for 30 minutes at 4°C: PE-like anti-human mesothelin PE (BiotechR&D Systems, Minneapolis, USA), which recognises an epitope contained within residues Glu_296_-Gly_580_ of mature mesothelin, and FITC-anti-human GFAP FITC (eEbBioscience, MassachusettesMassachusetts, USA), which is a glial cell-specific marker [39] for 30 minutes,. The stained cells were then washed with FACS buffer (PBS+0.1%FBS) and acquired on a FACSAria flow cytometer (BD Biosciences, Stockholm, Sweden). Data was analysed using FlowJo software (Treestar Inc.).

### Plasma preparation, and isolation and TILs expansion from glioma tumor tissue

Plasma was removed following centrifugation of whole blood samples, and stored at -80°C. Glioma tumor tissue was harvested in the course of tumor surgery at the Department of Neuro-Oncology at Karolinska University Hospital, Stockholm. Tumor tissue was immediately transferred to Cellgro GMP Serum-free DC medium medium supplemented with 5% pooled human AB serum. Tumor tissue was dissected into fragments of approximately 1-2mm3 using a sterile scalpel, or processed into a tissue homogenate using the BD Medimachine. Tissue fragments of the cell suspension were washed twice with PBS and cultured in 24 well plates in Cellgro medium plus 5% pooled human AB serum supplemented with recombinant IL-2 (1000IU/ml), IL-15(10ng/ml) and IL-21 (10ng/ml) (Prospec, Ness-Ziona, Israel). Medium was changed as necessary. Irradiated (55Gy) feeder cells (allogeneic PBMCs) at the ratio of 1 (feeder cells):10 (TILs) was added on day 7. OKT3 (anti-CD3 monoclonal antibody, BioLegend, San Diego, CA) was used at 10ng/ml as TILs became visible under the microscope. TILs were transferred to 6-well plates; upon achieving > 70% confluence in the 24-well surface. They were further expanded in G-Rex flasks (Wilson Wolf, New Brighton, MN) using 30ng OKT3/mL and irradiated (55Gry) allogeneic feeder cells at the ratio of 1 (feeder cells):10 (TILs).

### Whole blood assay (WBA) and IFN-γ ELISA

Heparinised whole blood was diluted at a ratio of 1:1.5 with R10 medium (RPMI 1640 L-glutamine (2mM) containing antibiotics (penicillin,100IU/mL and streptomycin, 10mg/mL) and 10% FBS, and one of three different cytokine conditions: (i) without cytokines (medium only); (ii) IL-7 (10ng/ml) and IL-2 (1000II/ml) or (iii) IL-2 (1000IU/ml), IL-15 (10ng/ml) and IL-21 (10ng/ml). The diluted blood was transferred to a 96-well culture pre-coated with the following antigens: mesothelin precursor peptide pool, megakaryocyte-potentiating factor (MPF) or mesothelin anchor for seven days at 37°C with 5% CO_2_ as described previously [40, 41]. Phytohaemagglutinin (PHA) and OKT3 (anti-human CD3 monoclonal antibody, BioLegend, CA, USA) were used as positive control while medium served as negative control. Supernatants were harvested seven days later to quantify antigen-specific interferon gamma (IFN-γ) production by sandwich ELISA (MABTECH, Stockholm, Sweden).

### FASCIA assay to assess CD3+/CD4+/CD8+T-cell proliferation

After seven days of incubation, supernatants from the WBA plates were harvested and stored at -20°C for cytokine detection. The remaining contents of the WBA plate (blood cells) were pooled (for the duplicate wells), washed with PBS and stained for the flow cytometric assay for specific cell-mediated immune-response in activated whole blood (FASCIA) [42] with a cocktail of monoclonal antibodies: anti-CD3 FITC, anti-CD4 APC, anti-CD8 PerCP and anti-TCRγδ PE. After 15-minute incubation at 4°C, red blood cells were lysed with Pharm lysing buffer (BD Biosciences, CA, USA) for 10 minutes followed by a 5-minute incubation at room temperature. Cells were then resuspended in PBS and acquired on a FACSCalibur flow cytometer (BD Biosciences, CA, USA). Analysis was done using the FlowJo software (Treestar, OR, USA). The proliferation ratio of the analysed cells was calculated based on the size and granularity of resting and activated cells (blasts) with the following formula: *PR = blast / (resting cells + blast)*. The stimulation percentage (SP_%_) was defined as a function of the proliferation ratio of the negative (medium) and positive (PHA) controls: *SP% = (PR_Ag_ - PR_medium_) / (PR_PHA_ - PR_medium_) x 100*.

### Intracellular cytokine staining

1.0 × 10^6^ PBMCs were stimulated with 1 μg/ml mesothelin peptide pool (Peptides & Elephants, Potsdam, Germany), medium control or PMA/Ionomycin (positive control, Sigma-Aldrich, St Louis, MI; USA) in R10 medium in the presence of Brefeldin A (10μg/mL, Sigma-Aldrich St Louis, MI, USA) for 6 hours at 37°C. Stimulation was stopped by transferring the cells to a 4°C refrigerator, followed by washing with FACS buffer and staining with the following reagents: anti-CD3 Pacific blue (BD Biosciences, CA, USA), anti-CD4 PerCP-Cy5.5 (BD Biosciences, CA, USA) and anti-CD8 APC-Cy7 (BD Biosciences, CA, USA). After a 15-minute incubation at 4°C, the cells were washed and fixed with a Fix/Perm reagent (Beckman coulter, CA, USA), followed by a further 30-minute incubation at 4°C with an intracellular antibody mix (anti-TNFα APC (BD Biosciences CA, USA), anti-IFN-γ PE-Cy7 (BD Biosciences CA, USA), anti-IL-2 PE (BD biosciences CA, USA) and anti-IL-17 FITC (BioLegend, CA, USA)). The stained cells were then washed with FACS buffer and acquired on a FACSAria flow cytometer (BD Biosciences, Stockholm, Sweden). Data was analysed using FlowJo software (Treestar Inc.).

### Quantitative mesothelin-specific IgG ELISA

Plasma IgG antibodies specific for the mesothelin precursor were determined by an indirect ELISA method developed in house. Briefly, 96-well U-bottom Maxisorp plates (Nunc, Roskilde, Denmark) were coated with either human IgG (Sigma, USA) as a reference standard ranging from 15000 ng/ml to 117 ng/ml in a seven-point serial dilution (1:2 ratio) or 1 μg/ml of the mesothelin precursor antigen (R&D systems, Minneapolis, MN) in separate wells. The plate was incubated for one hour at 37°C and washed 3 times with wash buffer (PBS 0.05% + Tween 20), followed by blocking for 1 hours with PBS 2% + BSA 0.05% Tween 20 at room temperature (RT). After five washes, diluted patient plasma were added to the assay plate and incubated for a further two hours at RT. The plate was then washed five times, incubated with a secondary anti-human IgG monoclonal antibody (ALP conjugated, 1:1000 dilution, Mabtech, Stockholm, Sweden) for one hour at RT and washed five times thereafter. Para-nitrophenylphosphate (pNPP, Thermo Fisher Scientific, MA, USA) was added to the assay plate, followed by 45-minute incubation at RT in the dark. The reaction was stopped by adding 1N NaOH, and the optical density was measured at 405nm using a Vmax kinetic microplate reader (Molecular Devices).

### Quantitative mesothelin ELISA

The quantitative determination of human mesothelin concentration in plasma was performed using the human mesothelin immunoassay kit (R&D systems, Minneapolis, MN) according to the manufacturer's protocol. The range of the standard curve was between 10 and 0.156 ng/mL, as previously reported [43].

### WBA IFN-γ responses to mesothelin peptides (Mesothelin precursor mapping)

Whole blood was diluted 1:1.5 with RPMI and co-incubated in a pre-coated plate with a panel of 42 peptides (1μg/ml) and a mesothelin peptide mix (1μg/peptide/ml; Peptides & Elephants, Potsdam, Germany) at 37°C, 5% CO_2_ during 7 days. The mesothelin peptide pool is a customised product comprising 42 × 15-mer peptides without overlap covering the entire length of the mesothelin protein. Mesothelin peptide-specific T-cells response was then defined after harvest the supernatant by IFN-γ production, quantified by ELISA (Mabtech, Stockholm, Sweden). Absolute values of IFN-γ production (in pg/ml) as well as the normalised average percentage of recognition were graphed. The normalisation for average IFN-γ production was calculated as follows: ((total IFN-γ production (peptides 1 to 42)) / (IFN-γ production per peptide)) × 100.

### Statistical analysis

GraphPad Prism 6 software was used for statistical analysis of differences between patient groups or within each group with the Mann-Whitney U-test or the Wilcoxon test. A p value of 0.05 or less was considered significant.

## SUPPLEMENTARY MATERIALS FIGURES AND TABLES



## Abbreviations

GBM: glioblastoma multiforme, IDH-wildtype/-mutant/NOS
OD: oligodendroglioma or anaplastic oligodendroglioma, IDH-mutant and 1p/19q-codeleted/NOS
A: diffuse or anaplastic astrocytoma, IDH-wildtype/-mutant/NOS
TIL: tumour-infiltrating lymphocytes
GKS: Gamma Knife Surgery
EGFR: epidermal growth factor receptor
VEGF: vascular endothelial growth factor
WBA: whole blood assay
IFN-γ: interferon gamma
TNF-α: tumour necrosis factor alpha
IL-2: interleukin 2
IL-7: interleukin 7
IL-15: interleukin 15
IL-21: interleukin 21
ELISA: enzyme-linked immunosorbent assay
NaOH: sodium hydroxide
FBS: foetal bovine serum
CO_2_: carbon dioxide
RT: room temperature
TCR: T-cell receptor
PBMC: peripheral blood mononuclear cells
HLA: human leukocyte antigen
MPF: megakaryocyte-potentiating factor
GPI: glycophosphatidylinositol GPI
PHA: Phytohaemagglutinin
PMA: phorbol 12-myristate 13-acetate
ALP: alkaline phosphatase
FASCIA: Flow cytometric assay for specific cell-mediated immune response in activated whole blood
CAR: chimeric antigen receptor
WHO: World Health Organisation
