# Supplementary material for: Mesothelin as a novel biomarker and immunotherapeutic target in human glioblastoma
Source: Oncotarget. 2017 Aug 16;8(46):80208–22. doi: 10.18632/oncotarget.20303 (PMC5655191; doi:10.18632/oncotarget.20303)
Supplement: Supplementary file 1 [file oncotarget-08-80208-s001.pdf]

Mesothelin as a novel biomarker and immunotherapeutic target in human glioblastoma

SUPPLEMENTARY MATERIALS

Supplementary Figure 1

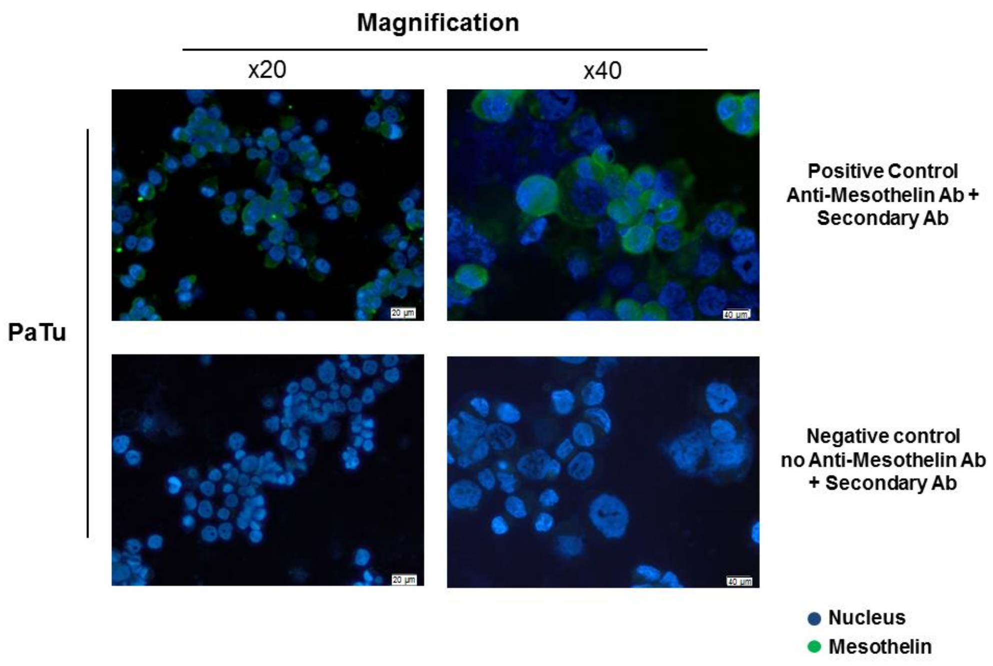

Supplementary Figure 1. Positive control for immunohistochemistry staining with pancreatic tumor cell line. The pancreatic cancer tumor cell line PaTu was fixed onto a microscope slide by cytospin and incubated overnight with rat monoclonal anti-human mesothelin antibody, 10µg/ml (R&D

systems, Minnesota, USA) at 4℃. After washing, slides were incubated for 30 minutes with Alexa Fluor 488-conjugated polyclonal goat anti-rat secondary antibody (Thermo Fisher Scientific, Massachusetts, USA) at 5μg/ml. The slides were then DAPI stained after washing, and mounted in mounting medium containing anti-fade. The stained control slides were examined and photographed with a camera-equipped microscope (Olympus BX51, Tokyo, Japan).

Supplementary Figure 2

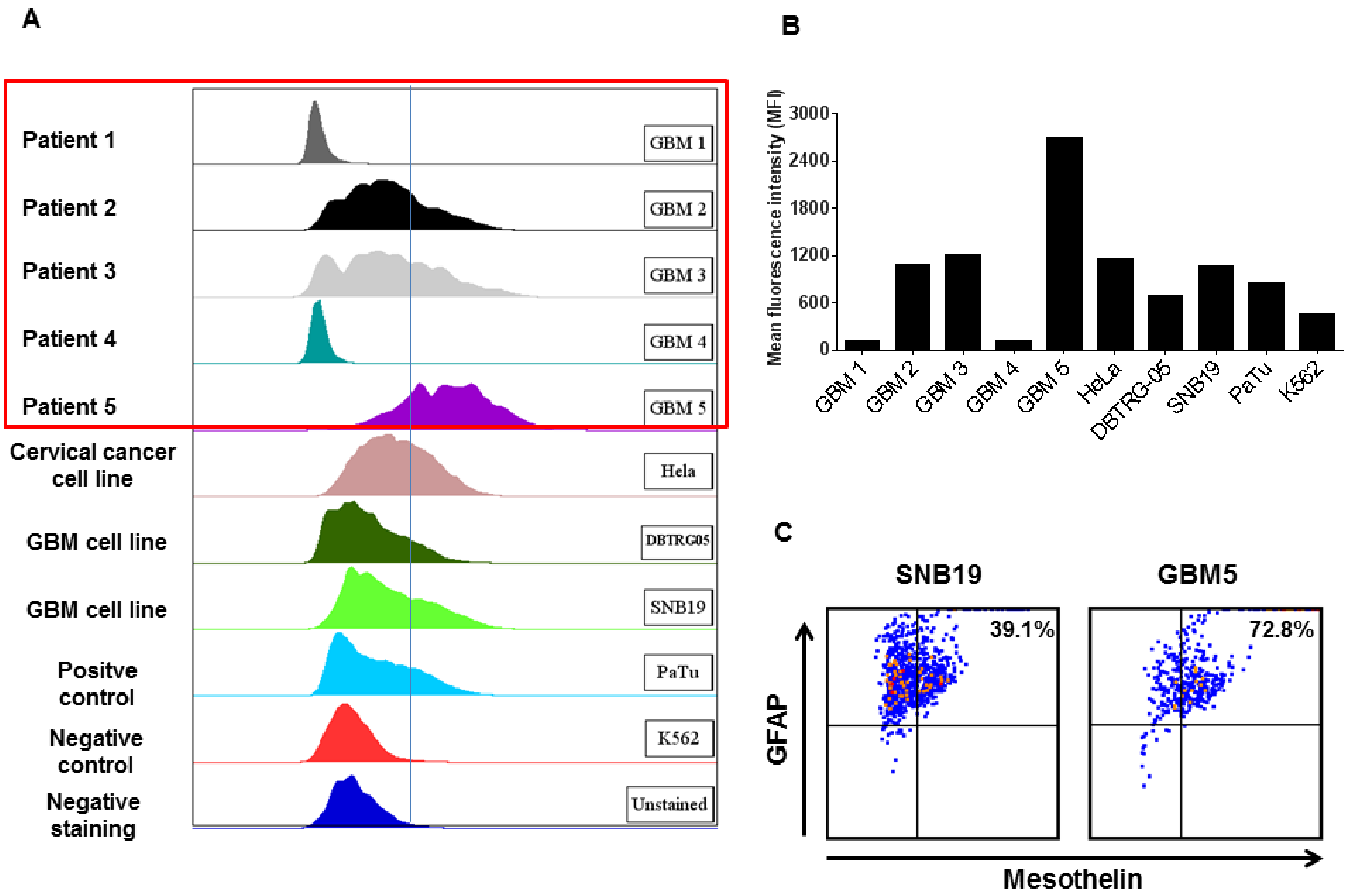

Supplementary Figure 2. Mesothelin expression on the surface of GBM cells. Cultured tumor cells from GBM tissue (isolated from five patients) were stained with an anti-human mesothelin monoclonal antibody conjugated to PE (R&D Systems, Minneapolis, USA) and acquired on a FACSAria. As controls, two different commercially available human GBM cell lines (SNB19 and DNTRG05), a positive control cell line (pancreatic cancer cell line PaTu), a negative control cell line (chronic myelogenous leukemia cell line K562) as well as the cervical cancer cell line HeLa were stained with the same antibody. (A) Flow cytometric analysis of the surface expression of mesothelin on the different cancer cell types, including the GBM cells from five patients (denoted as GBM1-5). Tumor cells from three out of five patients displayed strong surface expression of mesothelin. (B) Mean fluorescence intensity (MFI) values of mesothelin expression to indicate the number of individual mesothelin molecules present on the cell surface of the respective samples (primary GBM cells, GBM cell lines, control tumor cell lines). (C) Mesothelin and Glial fibrillary acidic protein (GFAP) co-staining of GBM cells from patient no.5 (GBM5) as well as a GBM cell line (SNB19) to show that tumor cells of glial origin (GFAP+)[47] highly co-express mesothelin.

Supplementary Figure 3A

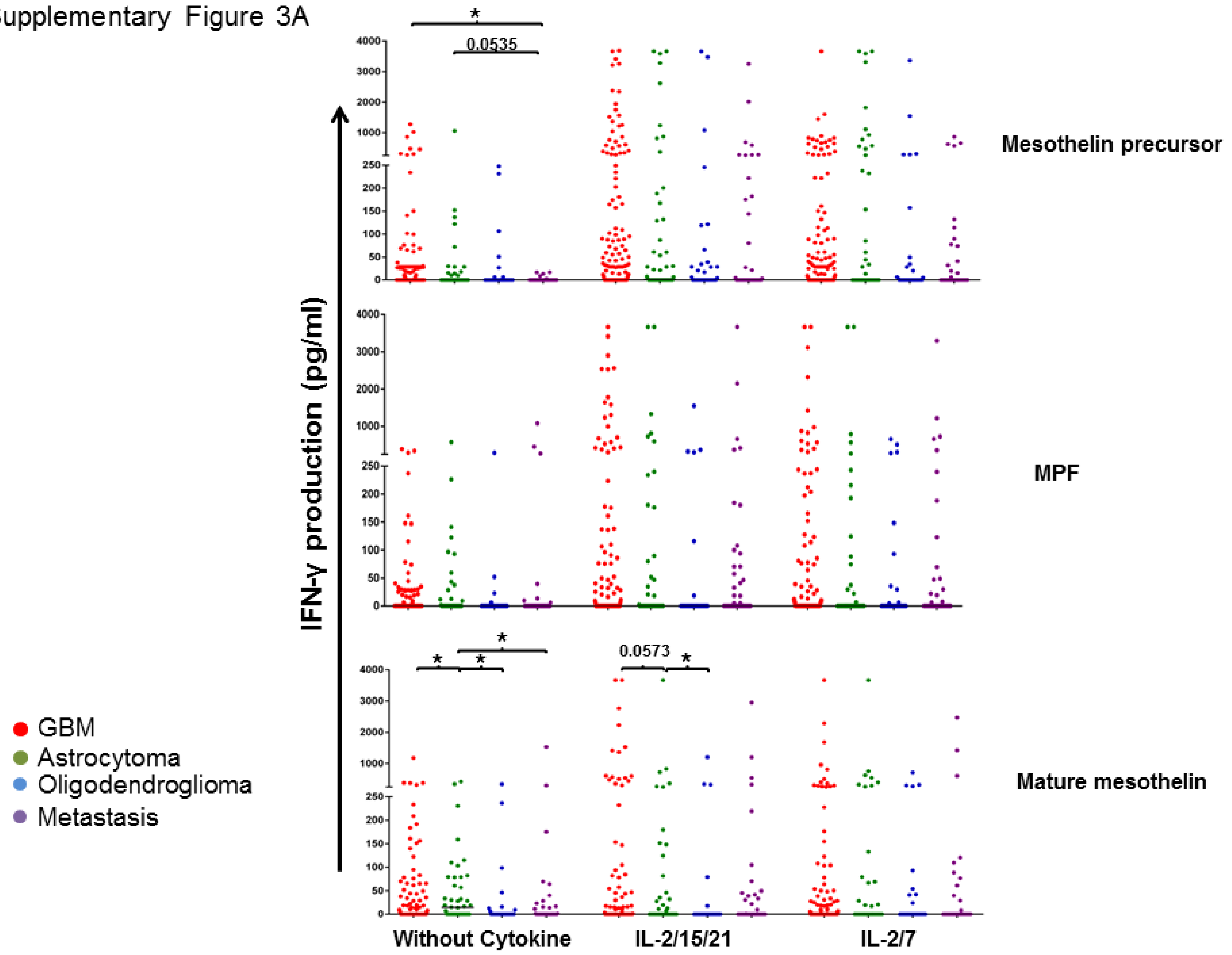

Supplementary Figure 3B

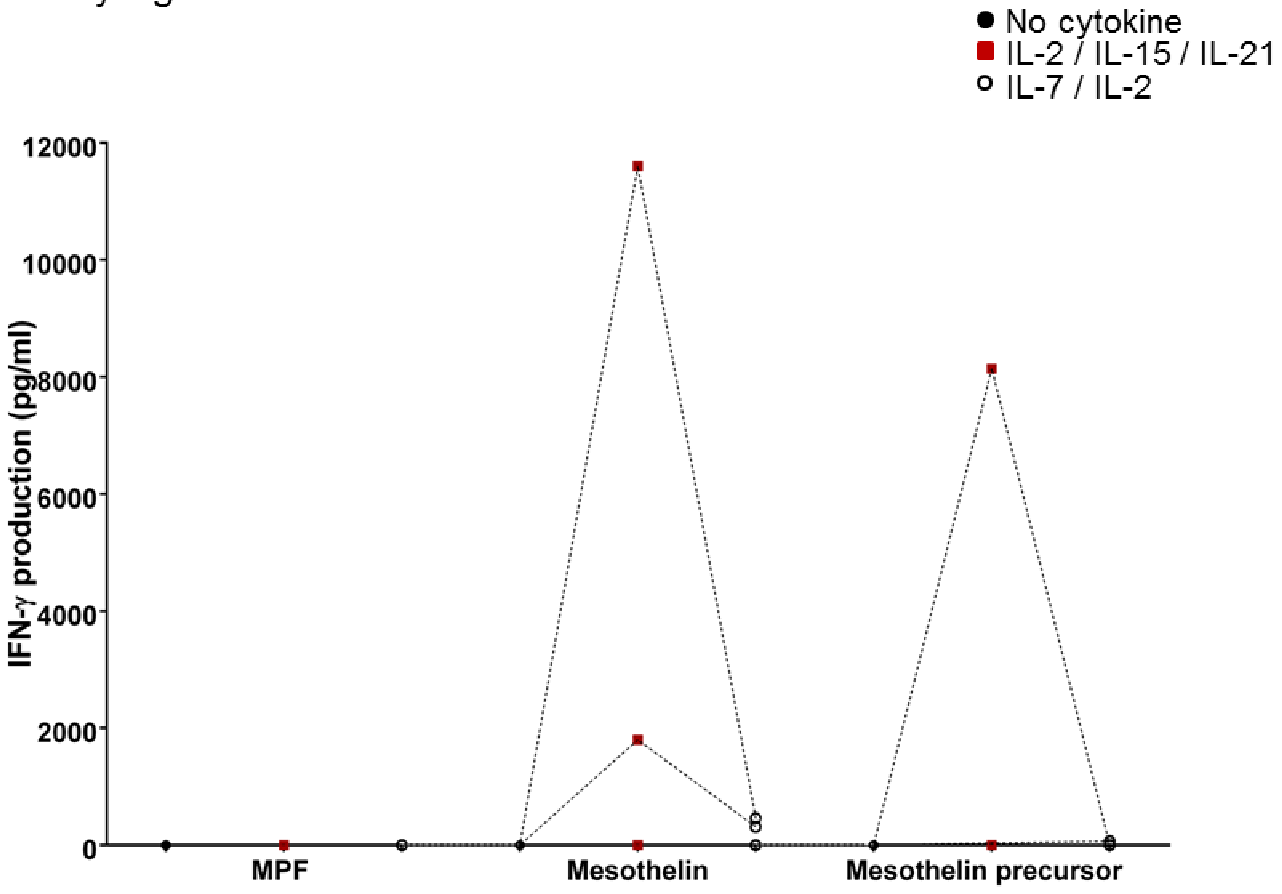

PBMCs from three healthy donors

Supplementary figure 3. Comparison of whole-blood IFN-γ responses between groups of patients with different diagnoses of malignant glioma to the mesothelin precursor protein, MPF and mesothelin component with or without cytokine conditioning. Whole-blood obtained from patients with malignant glioma (GBM, astrocytoma, OD, brain metastasis) (A) or healthy donors (B) were cultured with the mesothelin precursor molecule, the MPF component or mature mesothelin in the absence of cytokine conditioning, with IL-2/IL-7 or IL-2/IL-15/IL-21 conditioning over seven days. A control test with peripheral blood from three healthy donors was also performed. Supernatants were then harvested for IFN-γ detection by ELISA. Shown are dot plots representing responses of individual patients with the respective diagnoses. Although a general upregulation of IFN-γ production in response to stimulation with mesothelin peptides was seen with the IL-2/IL-15/IL-21 combination, significant difference between the patient groups was only visible in the absence of cytokine conditioning. In healthy donors, peripheral blood IFN-γ response to mesothelin was below detection levels without cytokine conditioning, but was dramatically increased with IL-2/IL-15/IL-21 conditioning. Mann-Whitney test of medians was performed to gauge statistical significance. *$p<0.05$; **$p<0.001$.

Supplementary Figure 4

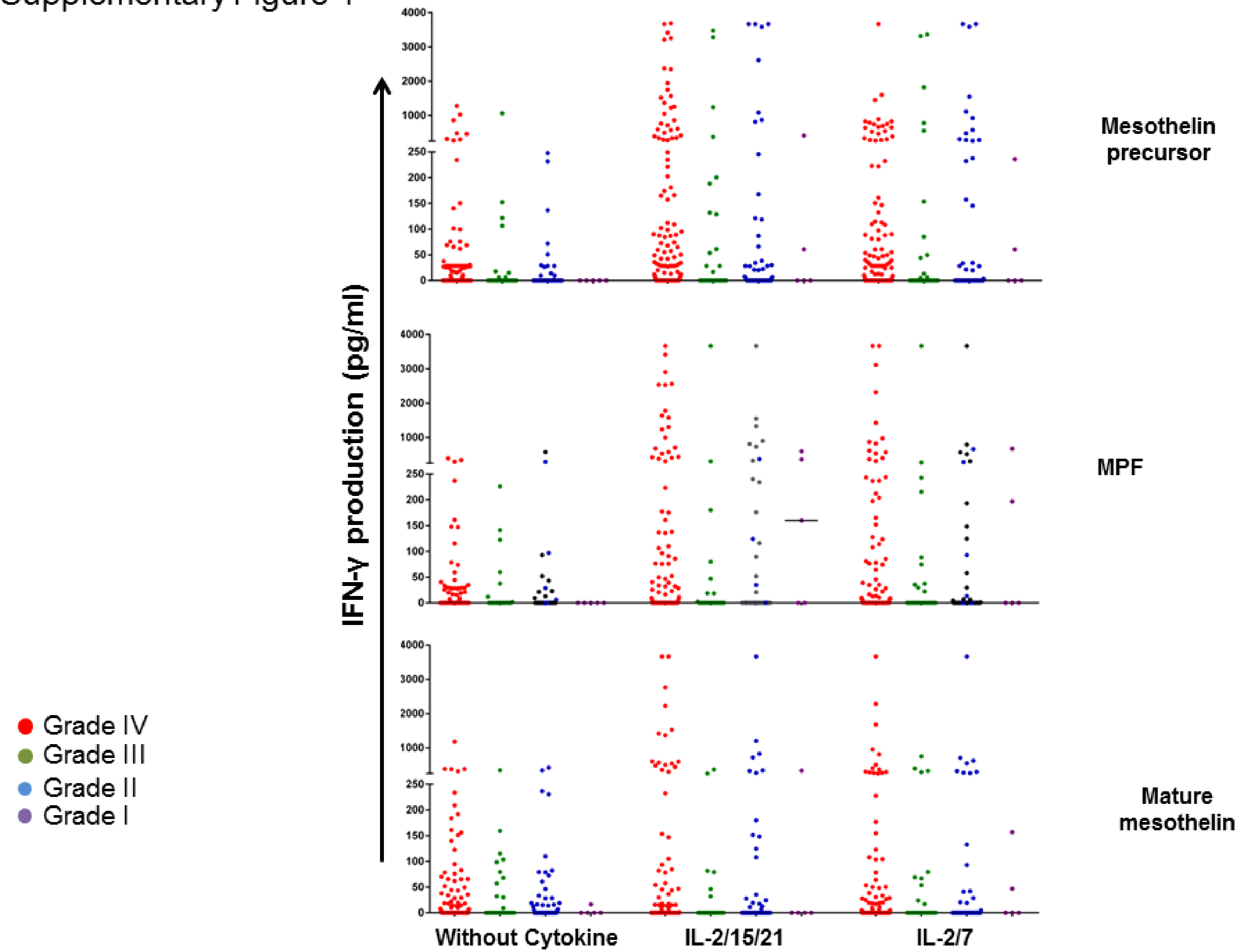

Supplementary Figure 4. Comparison of whole-blood IFN-γ responses to the mesothelin precursor protein, MPF and mesothelin component with or without cytokine conditioning between groups of patients with different WHO grades of CNS tumors. Whole-blood obtained from patients with different WHO grades of CNS tumors/malignant glioma (grades 1-IV) were cultured with the mesothelin precursor molecule (A), the MPF component (B) or mature mesothelin (C) in the absence of cytokine conditioning, with IL-2/IL-7 or IL-2/IL-15/IL-21 conditioning over seven days. Supernatants were then harvested for IFN-γ detection by ELISA. Shown are dot plots representing responses of individual patients with the respective diagnoses. Mann-Whitney test of medians was performed to gauge statistical significance. *$p<0.05$; **$p<0.001$.

Supplementary Figure 5

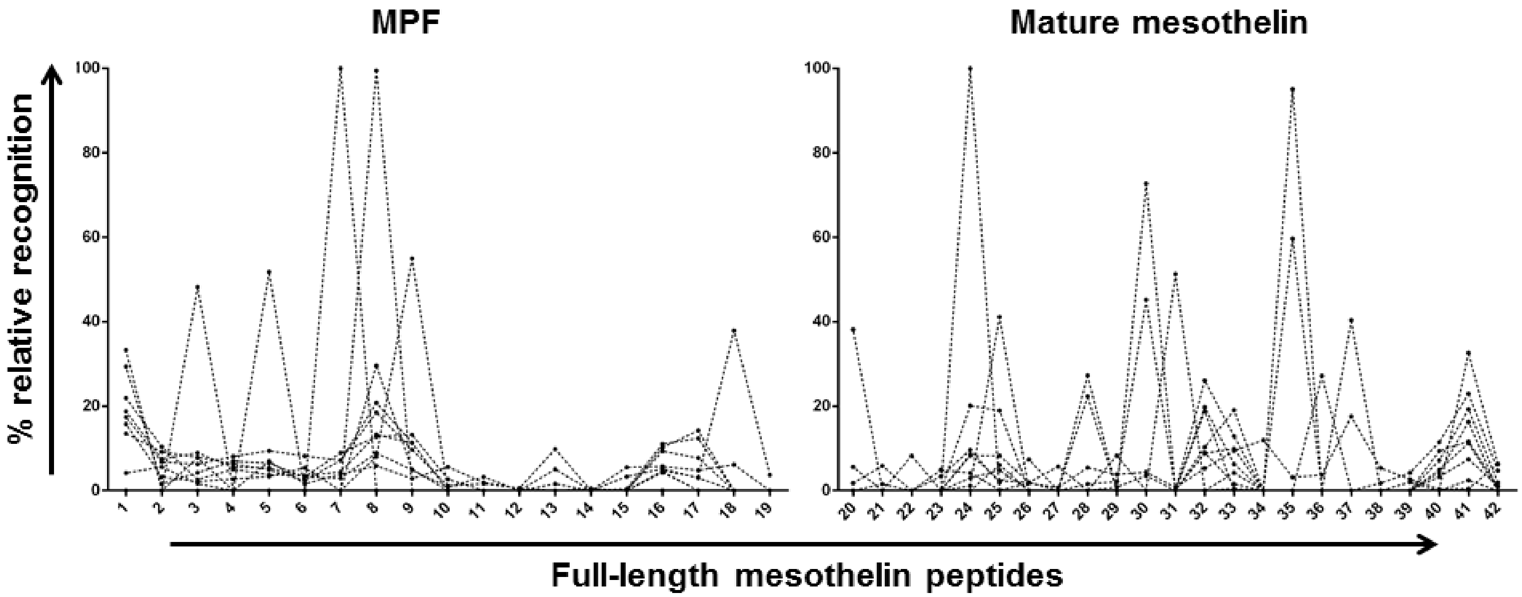

Supplementary Figure 5. Recognition of mesothelin peptides by individual patients with GBM defined by IFN-γ production in whole blood assays. Whole-blood obtained from patients GBM were cultured with a peptide mix (42 peptides) spanning the mesothelin precursor molecule; the first 19 peptides corresponding to the MPF component (B) and the remaining 23 peptides corresponding to mature mesothelin in the absence of cytokine conditioning of whole blood. The assay was performed over a seven-day period. Supernatants were then harvested for IFN-γ detection by ELISA. Shown are dot plots with connecting lines representing responses of individual patients to each of the mesothelin precursor peptides. The percentage recognition of the peptides per patient was calculated as follows: **((IFN-γ production to peptide/IFN-γ production to entire mesothelin precursor peptide mix)/100)**. The resulting percentage denotes the strength of the cellular immune response to the designated peptide within the mesothelin precursor molecule. Immune recognition hotspots are found mainly in the mesothelin component, with one particular patient exhibiting very strong responses. Mann-Whitney test of medians was performed to gauge statistical significance. *$p<0.05$; **$p<0.001$.
